# Assessment of Neonatal Intensive Care Unit Practices, Morbidity, and Mortality Among Very Preterm Infants in China

**DOI:** 10.1001/jamanetworkopen.2021.18904

**Published:** 2021-08-02

**Authors:** Yun Cao, Siyuan Jiang, Jianhua Sun, Mingyan Hei, Laishuan Wang, Huayan Zhang, Xiaolu Ma, Hui Wu, Xiaoying Li, Huiqing Sun, Wei Zhou, Yuan Shi, Yanchen Wang, Xinyue Gu, Tongling Yang, Yulan Lu, Lizhong Du, Chao Chen, Shoo K. Lee, Wenhao Zhou

**Affiliations:** 1 Division of Neonatology, Children’s Hospital of Fudan University, Shanghai, China; 2 Division of Neonatology, Shanghai Children’s Medical Center, Shanghai Jiao Tong University School of Medicine, Shanghai, China; 3 Neonatal Center, Beijing Children's Hospital, Capital Medical University, Beijing, China; 4 Division of Neonatology, Division of Neonatology and Center for Newborn Care, Guangzhou Women and Children’s Medical Center, Guangdong, China; 5 Department of Pediatrics, Children’s Hospital of Philadelphia and University of Pennsylvania Perelman School of Medicine, Philadelphia; 6 Division of Neonatology, The Children’s Hospital Zhejiang University School of Medicine, Zhejiang, China; 7 Division of Neonatology, The First Bethune Hospital of Jilin University, Jilin, China; 8 Division of Neonatology, Qilu Children’s Hospital of Shandong University, Shandong, China; 9 Division of Neonatology, Children’s Hospital Affiliated with Zhengzhou University, Children’s Hospital of Henan Zhengzhou, Hennan, China; 10 Division of Neonatology, Children’s Hospital of Chongqing Medical University, Chongqing, China; 11 NHC Key Laboratory of Neonatal Diseases, Fudan University, Children’s Hospital of Fudan University, Shanghai, China; 12 Center for Molecular Medicine, Pediatrics Research Institute, Children’s Hospital of Fudan University, Shanghai, China; 13 Maternal-Infant Care Research Centre and Department of Pediatrics, Mount Sinai Hospital, Toronto, Ontario, Canada; 14 Department of Pediatrics, University of Toronto, Toronto, Ontario, Canada; 15 Department of Obstetrics and Gynecology and Dalla Lana School of Public Health, University of Toronto, Toronto, Ontario, Canada

## Abstract

**Importance:**

The Chinese Neonatal Network was established in 2018 and maintains a standardized national clinical database of very preterm or very low-birth-weight infants in tertiary neonatal intensive care units (NICUs) throughout China. National-level data on outcomes and care practices of very preterm infants (VPIs) in China are lacking.

**Objective:**

To assess the care practices in NICUs and outcomes among VPIs in China.

**Design, Setting, and Participants:**

A cohort study was conducted comprising 57 tertiary hospitals from 25 provinces throughout China. All infants with gestational age (GA) less than 32 weeks who were admitted to the 57 NICUs between January 1 and December 31, 2019, were included.

**Main Outcomes and Measures:**

Care practices, morbidities, and survival were the primary outcomes of the study. Major morbidities included bronchopulmonary dysplasia, severe intraventricular hemorrhage (grade ≥3) and/or periventricular leukomalacia, necrotizing enterocolitis (stage ≥2), sepsis, and severe retinopathy of prematurity (stage ≥3).

**Results:**

A total of 9552 VPIs were included, with mean (SD) GA of 29.5 (1.7) weeks and mean (SD) birth weight of 1321 (321) g; 5404 infants (56.6%) were male. Antenatal corticosteroids were used in 75.6% (6505 of 8601) of VPIs, and 54.8% (5211 of 9503)were born through cesarean delivery. In the delivery room, 12.1% of VPIs received continuous positive airway pressure and 26.7% (2378 or 8923) were intubated. Surfactant was prescribed for 52.7% of the infants, and postnatal dexamethasone was prescribed to 9.5% (636 of 6675) of the infants. A total of 85.5% (8171) of the infants received complete care, and 14.5% (1381) were discharged against medical advice. The incidences of the major morbidities were bronchopulmonary dysplasia, 29.2% (2379 of 8148); severe intraventricular hemorrhage and/or periventricular leukomalacia, 10.4% (745 of 7189); necrotizing enterocolitis, 4.9% (403 of 8171 ); sepsis, 9.4% (764 of 8171); and severe retinopathy of prematurity, 4.3% (296 of 6851) among infants who received complete care. Among VPIs with complete care, 95.4% (7792 of 8171) survived: 65.6% (155 of 236) at 25 weeks’ or less GA, 89.0% (880 of 988) at 26 to 27 weeks’ GA, 94.9% (2635 of 2755)at 28 to 29 weeks’ GA, and 98.3% (4122 of 4192) at 30 to 31 weeks’ GA. Only 57.2% (4677 of 8171) of infants survived without major morbidity: 10.5% (25 of 236) at 25 weeks’ or less GA, 26.8% (48 of 179) at 26 to 27 weeks’ GA, 51.1% (1409 of 2755) at 28 to 29 weeks’ GA, and 69.3% (2904 of 4192) at 30 to 31 weeks’ GA. Among all infants admitted, the survival rate was 87.6% (8370 of 9552)and survival without major morbidities was 51.8% (4947 of 9552).

**Conclusions and Relevance:**

The findings of this study suggest that survival and survival without major morbidity of VPIs in Chinese NICUs have improved but remain lower than in high-income countries. Comprehensive and targeted quality improvement efforts are needed to provide complete care for all VPIs, optimize obstetrical and neonatal care practices, and improve outcomes.

## Key Points

**Question:**

What are the care practices and outcomes for very preterm infants in Chinese neonatal intensive care units?

**Findings:**

In this cohort study of 9552 very preterm infants from 57 tertiary neonatal intensive care units throughout China in 2019, 86% received complete care, among whom 95% survived and 57% survived without major morbidities. Only 76% of the infants received antenatal corticosteroids, and 12% of the infants received delivery room continuous positive airway pressure.

**Meaning:**

The findings of this study suggest that survival and survival without major morbidity of very preterm infants in Chinese neonatal intensive care units remain lower than in high-income countries and clinical quality improvement as well as systems and health services reorganization are needed to improve outcomes.

## Teaser

This cohort study evaluates the care practices used for as well as the survival and major morbidities among very preterm infants hospitalized in neonatal intensive care units in China.

## Introduction

In recent decades, there have been major advances in perinatal and neonatal intensive care in China.^1,2^ Neonatal mortality has been reduced substantially, but preterm birth has become the leading cause of neonatal death.^3^ There are approximately 0.2 million very preterm infants (VPIs) (<32 weeks’ gestational age [GA]) born every year in China.^4,5^ Although steady improvements in outcomes have been reported, VPIs in China continue to contribute disproportionately to the burden of neonatal death and long-term developmental disability. Currently, national-level data on outcomes and care practices for VPIs in China are lacking. Information on care practices, morbidity, and mortality of VPIs is essential to benchmark outcomes for institutions to evaluate their performance, identify deficiencies in care, facilitate quality improvement, improve health services delivery, and support parental counseling and clinical decision-making.

The Chinese Neonatal Network (CHNN) was founded in 2018 and has established a standardized national clinical database of VPIs in tertiary neonatal intensive care units (NICUs) throughout China to monitor outcomes and changes in care practices and explore potential strategies for improving neonatal care. The CHNN database was launched with prospective data collection starting from January 1, 2019. To our knowledge, the present study is the first full-year report of the CHNN database assessing the outcomes and care practices of VPIs in Chinese NICUs in 2019, aiming to profile the current state of care and outcomes for VPIs in Chinese tertiary NICUs.

## Methods

### Design

The CHNN hospitals are tertiary referral institutions with large neonatal services and recognized expertise in caring for high-risk neonates and were selected to be representative of different regions of the country. A total of 58 hospitals from 25 provinces throughout China participated in the CHNN in 2019 (eFigure in the Supplement), caring for approximately 5% of all VPIs in China. These hospitals included all government-designated neonatal centers of excellence in China, including 4 national children’s medical centers, 5 regional children’s medical centers, and 30 provincial perinatal or children’s medical centers. The other 19 hospitals were major referral centers in large cities across China. Fifty-seven hospitals collected whole-year data of VPIs admitted to their NICUs in 2019 and were enrolled in this study (1 hospital was excluded because of incomplete data for logistical reasons but will be included in future studies). Forty-three hospitals were perinatal centers with birthing facilities, and 14 hospitals were freestanding children’s hospitals that admitted only outborn infants. The median number of NICU beds was 40 (interquartile range [IQR], 30-62), and the median number of intermediate-level and continuing care neonatal beds was 66 (IQR, 40-91). For perinatal centers, the median number of annual deliveries was 10 280 (IQR, 6273-15 423). The median number of full-time equivalent neonatologists was 19 (IQR, 12-27), and the median number of NICU nurses was 42 (IQR, 30-65). All hospitals had facilities to provide long-term invasive or noninvasive ventilation support. General surgery was available in 47 hospitals, patent ductus arteriosus ligation in 45 hospitals, and cardiac surgeries requiring extracorporeal circulation in 35 hospitals. All hospitals could perform bedside head ultrasonography, and 55 hospitals could perform magnetic resonance imaging examination. All hospitals performed onsite screening for retinopathy of prematurity (ROP), and 47 performed ROP treatment. All hospitals provided mother’s milk for feeding, and 20 hospitals provided donor milk.

This study was approved by the ethics review board of Children’s Hospital of Fudan University, which was recognized by all participating hospitals. Waiver of consent was granted at all sites owing to the use of deidentified patient data. This study followed the Strengthening the Reporting of Observational Studies in Epidemiology (STROBE) reporting guideline for cohort studies.

This was a hospital-based cohort. The CHNN database includes all infants with less than 32 weeks’ GA or with birth weight less than 1500 g and admitted to participating NICUs. Stillbirths, delivery room deaths, and infants transferred to nonparticipating hospitals within 24 hours after birth are not captured by the database. Readmissions and transfers between participating hospitals were tracked as data from the same infants. Infants were followed up until NICU discharge or transfer or death. In this study, we included all infants with less than 32 weeks’ GA and admitted to participating NICUs between January 1 and December 31, 2019, without exclusion.

Trained data abstractors were responsible for data acquisition in each hospital. Data were directly entered into a customized database with built-in error checking and a standard manual of operations and definitions. Data were electronically transmitted to the CHNN coordinating center in Children’s Hospital of Fudan University with patient identity kept confidential. Quarterly data checks were performed by the coordinating center for quality and completeness. Quarterly site-specific data quality reports were given to each site, and data records were returned for corrections if needed. Data quality audit using data reabstraction was performed annually. Site investigators were responsible for data quality control in each site.

### Outcomes

Care practices were chosen based on their association with neonatal outcomes or because they were important indicators of quality of care. The care practices included antenatal corticosteroids, antenatal antibiotics, cesarean delivery, delivery room resuscitation, surfactant therapy, respiratory support, postnatal corticosteroids, and breast milk feeding. Antenatal corticosteroids were defined as a partial or complete course of antenatal corticosteroids. Surfactants included those used both in delivery rooms and in NICUs. Postnatal corticosteroids referred to intravenous dexamethasone used for bronchopulmonary dysplasia (BPD). Invasive ventilation included high-frequency and conventional ventilation via endotracheal tubes. Noninvasive respiratory support included noninvasive high-frequency ventilation, noninvasive positive pressure ventilation, nasal continuous positive airway pressure, and high-flow oxygen.

Morbidities included intraventricular hemorrhage (IVH) (grade ≥3) and/or cystic periventricular leukomalacia (PVL), necrotizing enterocolitis (NEC) (stage ≥2), sepsis, ROP (stage ≥3), and BPD. Intraventricular hemorrhage was defined as greater than or equal to grade 3 according to the Papile criteria.^6^ Cystic PVL was defined as the presence of periventricular cysts identified on cranial ultrasonography or magnetic resonance imaging. Necrotizing enterocolitis was defined according to Bell criteria.^7,8^ Sepsis was defined as positive blood or cerebrospinal fluid culture and antibiotic therapy or intent of antibiotic therapy for 5 days or longer.^9^ Retinopathy of prematurity was diagnosed according to the International Classification of Retinopathy of Prematurity.^10^ Bronchopulmonary dysplasia was defined as ventilation or oxygen dependency at 36 weeks’ corrected age or at discharge, transfer, or death before 36 weeks.^11^ Survival and survival without major morbidities (IVH grade ≥3 or PVL, NEC stage ≥2, sepsis, ROP stage ≥3, and BPD) were studied.

A large proportion of VPIs were discharged against medical advice (DAMA), which meant parents terminated treatment before the treating physicians recommended discharge. DAMA substantially compromised the survival of VPIs and influenced the morbidities. The poorer outcomes of DAMA infants may be attributable to infant characteristics and perinatal care practices or early termination of appropriate medical treatment. Therefore, we studied morbidities and survival among infants with complete care, DAMA infants, and all admitted infants separately. Morbidities and survival among infants with complete care may reflect the quality of care more accurately by excluding the influence of termination of care against medical advice on the outcomes. Outcomes of all infants reflected the general survival status of all infants admitted to CHNN NICUs, regardless of whether care was terminated against medical advice. We used predefined criteria to make assumptions about survival of DAMA infants, as we were unable to determine the survival of these infants after discharge because of logistical constraints. If DAMA infants required invasive or noninvasive mechanical ventilation, inotropes infusion, or total parenteral nutrition (no enteral feeds initiated) on the day of discharge, we assumed that they would not survive after discharge.

Gestational age was determined using the hierarchy of best obstetric estimate based on prenatal ultrasonography, menstrual history, obstetric examination, or all 3 factors. If the obstetric estimate was not available or was different from the postnatal estimate of gestation by more than 2 weeks, the GA was estimated using the Ballard score.^12^ Small for gestational age was defined as birth weight less than the 10th percentile for the GA according to the Chinese neonatal birth weight values.^13^ Prenatal care was defined as 1 or more pregnancy-related hospital visit during pregnancy. The Transport Risk Index of Physiologic Stability score^14,15^ was used as an illness severity score on NICU admission.

### Statistical Analysis

Demographic variables, care practices, morbidities, and mortality are summarized using descriptive statistics without comparison. Statistical results are presented by frequency (percentages) for categorical variables and means (SDs) or medians (IQRs) for continuous variables. The results are displayed by each week of GA. Survival rates and morbidities of DAMA and complete-care infants were compared using χ^2^ tests. Statistical analysis was performed using Stata, version 15.0 (StataCorp LLC).

## Results

A total of 9552 VPIs with the mean (SD) GA of 29.5 (1.7) weeks and the mean (SD) birth weight of 1321 (321) g composed our study population, after excluding 1271 infants with GA greater than or equal to 32 weeks from the total 2019 CHNN cohort of 10 823 infants, which enrolled all infants admitted to 57 participating NICUs with gestational age less than 32 weeks or birth weight less than 1500 g.

Among 9552 VPIs included in our study, only 3.7% (353) were 25 weeks’ GA or less and 13.5% (1293) were 26 to 27 weeks’ GA. Most infants were 28 to 31 weeks’ GA (Table 1). A total of 56.6% (5404) of VPIs were male, 43.4% (4148) were female, 30.0% (2870 of 9552) were multiple births, and 36.5% (3482 of 9552) were outborn. The mean maternal age was 30.8 (5.0) years. Overall, 99.0% (9114 of 9209) of mothers received prenatal care, 18.8% (1761 of 9372) were diagnosed with hypertension, and 17.1% (1600 of 9353) were diagnosed with any type of diabetes.

**Table 1.** Infant and Maternal Characteristics of Very Preterm Infants (<32 Weeks' Gestation) Admitted to 57 Chinese Neonatal Intensive Care Units

Characteristic	No./total No. (%)									
	Total	Gestational age, wk								
		≤23	24	25	26	27	28	29	30	31
**Infant characteristics**										
No.	9552	32	92	229	451	842	1424	1783	2169	2530
Gestational age, mean (SD), wk	29.5 (1.7)	23.1 (0.7)	24.5 (0.3)	25.5 (0.3)	26.4 (0.3)	27.5 (0.3)	28.4 (0.3)	29.4 (0.3)	30.4 (0.3)	31.4 (0.3)
Birth weight, mean (SD), g	1321 (321)	576 (107)	716 (116)	825 (126)	918 (137)	1046 (154)	1160 (188)	1297 (219)	1441 (254)	1568 (288)
Sex										
Male	5404/9545 (56.6)	22/32 (68.8)	60/92 (65.2)	117/229 (51.1)	261/451 (57.9)	499/842 (59.3)	842/1424 (59.1)	1016/1783 (57.0)	1205/2168 (55.6)	1382/2524 (54.8)
Female	4141/9545 (43.4)	10/32 (31.2)	32/92 (34.8)	112/229 (48.9)	190/451 (42.1)	343/842 (40.7)	582/1424 (40.9)	767/1783 (43.0)	963/2168 (44.4)	1142/2524 (45.2)
Small for gestational age, No. (%)	651 (6.8)	NA	2 (2.2)	3 (1.3)	14 (3.1)	16 (1.9)	61 (4.3)	104 (5.8)	176 (8.1)	275 (10.9)
Multiple birth, No. (%)	2870 (30.0)	16 (50.0)	32 (34.8)	84 (36.7)	174 (38.6)	270 (32.1)	429 (30.1)	529 (29.7)	611 (28.2)	725 (28.7)
Outborn, No. (%)	3482 (36.5)	12 (37.5)	21 (22.8)	77 (33.6)	192 (42.6)	348 (41.3)	550 (38.6)	637 (35.7)	758 (34.9)	887 (35.1)
1-min Apgar score ≤3	603/9252 (6.5)	16/31 (51.6)	31/90 (34.4)	33/217 (15.2)	47/431 (10.9)	74/809 (9.1)	105/1369 (7.7)	115/1738 (6.6)	100/2111 (4.7)	82/2456 (3.3)
5-min Apgar score ≤3	120/8945 (1.3)	2/27 (7.4)	5/86 (5.8)	11/201 (5.5)	14/416 (3.4)	16/782 (2.0)	16/1319 (1.2)	22/1678 (1.3)	23/2051 (1.1)	11/2385 (0.5)
TRIPS score, median (IQR)^a^	13 (6-19)	28 (21-44)	22 (14-30)	21 (12-28)	19 (11-28)	15 (8-22)	13 (6-21)	12 (6-19)	12 (6-15)	8 (5-13)
Major birth defect, No. (%)	78 (0.8)	0	0	1 (0.4)	2 (0.4)	5 (0.6)	9 (0.6)	20 (1.1)	16 (0.7)	25 (1.0)
**Maternal characteristics**										
Age, mean (SD), y	30.8 (5.0)	31.8 (5.9)	31.4 (3.9)	32.0 (4.7)	31.1 (4.5)	31.3 (4.9)	30.9 (5.1)	30.6 (4.9)	30.8 (5.0)	30.6 (5.1)
≥1 Prenatal visit	9114/9209 (99.0)	29/29 (100)	88/90 (97.8)	221/222 (99.5)	434/436 (99.5)	792/798 (99.2)	1344/1363 (98.6)	1710/1725 (99.1)	2072/2099 (98.7)	2424/2447 (99.1)
Hypertension	1761/9372 (18.8)	1/32 (3.1)	7/91 (7.7)	10/222 (4.5)	40/445 (9.0)	102/817 (12.5)	224/1386 (16.2)	328/1751 (18.7)	462/2131 (21.7)	587/2497 (23.5)
Diabetes	1600/9353 (17.1)	1/32 (3.1)	13/92 (14.1)	30/221 (13.6)	82/444 (18.5)	124/810 (15.3)	265/1386 (19.1)	299/1750 (17.1)	380/2128 (17.9)	406/2490 (16.3)
Primigravida	4438/9486 (46.8)	16/32 (50.0)	56/92 (60.9)	116/228 (50.9)	229/445 (51.5)	403/835 (48.3)	663/1413 (46.9)	820/1775 (46.2)	953/2157 (44.2)	1182/2509 (47.1)

Abbreviations: IQR, interquartile range; NA, not applicable; NICU, neonatal intensive care unit; TRIPS, Transport Risk Index of Physiologic Stability.

^a^ TRIPS scores range from 0 to 54. Higher values represent greater illness severity.

### Care Practices

Overall, 75.6% (6505 of 8601) of infants received at least 1 dose of antenatal corticosteroids, and the incidence of use increased with GA (Table 2): 37.0% (10 of 27) at 23 weeks’ GA or less, 68.1% (196 of 288) at 24 to 25 weeks’ GA, 73.7% (825 of 1135) at 26 to 27 weeks’ GA, and 76.5% (5474 of 7151) at 28 to 31 weeks’ GA. Cesarean birth was the mode of delivery for 54.8% (5211 of 9503) of all infants, but only 17.1% (60 of 351) for infants 25 weeks’ GA or less and 31.0% (398 of 1283) for infants 26 to 27 weeks’ GA.

**Table 2.** Care Practices for Very Preterm Infants (<32 Weeks' Gestation) Admitted to 57 Chinese NICUs

Variable	Infants, No./total No. (%)									
	Total	Gestational age, wk								
		≤23	24	25	26	27	28	29	30	31
No.	9552	32	92	229	451	842	1424	1783	2169	2530
Antenatal corticosteroids	6505/8601 (75.6)	10/27 (37.0)	54/86 (62.8)	142/202 (70.3)	276/396 (69.7)	549/739 (74.3)	974/1274 (76.5)	1232/1616 (76.2)	1494/1965 (76.0)	1774/2296 (77.3)
Antenatal antibiotics	3614/7935 (45.5)	14/25 (56.0)	38/84 (45.2)	84/189 (44.4)	162/358 (45.3)	294/655 (44.9)	565/1184 (47.7)	661/1470 (45.0)	851/1846 (46.1)	945/2124 (44.5)
Cesarean delivery	5211/9503 (54.8)	4/32 (12.5)	12/92 (13.0)	44/227 (19.4)	101/448 (22.5)	297/835 (35.6)	722/1407 (51.3)	996/1779 (56.0)	1358/2160 (62.9)	1677/2523 (66.5)
Delivery room resuscitation										
CPAP	1080/8923 (12.1)	2/30 (6.7)	12/89 (13.5)	37/216 (17.1)	64/413 (15.5)	100/770 (13.0)	175/1325 (13.2)	210/1670 (12.6)	217/2030 (10.7)	263/2380 (11.1)
Intubation	2378/8923 (26.7)	27/30 (90.0)	70/89 (78.7)	132/216 (61.1)	199/413 (48.2)	339/770 (44.0)	433/1325 (32.7)	435/1670 (26.0)	412/2030 (20.3)	331/2380 (13.9)
Chest compression	325/8923 (3.6)	3/30 (10.0)	14/89 (15.7)	17/216 (7.9)	24/413 (5.8)	39/770 (5.1)	53/1325 (4.0)	56/1670 (3.4)	66/2030 (3.3)	53/2380 (2.2)
Epinephrine	201/8923 (2.3)	3/30 (10.0)	10/89 (11.2)	13/216 (6.0)	14/413 (3.4)	27/770 (3.5)	26/1325 (2.0)	41/1670 (2.5)	39/2030 (1.9)	28/2380 (1.2)
Delayed cord clamping	1446/6354 (22.8)	1/24 (4.2)	15/74 (20.3)	22/164 (13.4)	52/295 (17.6)	115/542 (21.2)	197/971 (20.3)	295/1194 (24.7)	337/1408 (23.9)	412/1682 (24.5)
Respiratory treatment^a^										
Surfactant therapy	3518/6675 (52.7)	2/2 (100)	17/18 (94.4)	79/89 (88.8)	173/214 (80.8)	350/477 (73.4)	618/923 (67.0)	794/1313 (60.5)	771/1647 (46.8)	714/1992 (35.8)
Postnatal corticosteroids^b^	636/6675 (9.5)	2/2 (100)	13/18 (72.2)	43/89 (48.3)	72/214 (33.6)	104/477 (21.8)	156/923 (16.9)	111/1313 (8.5)	75/1647 (4.6)	61/1992 (3.1)
Invasive ventilation^c^	2591/6675 (38.8)	2/2 (100)	16/18 (88.9)	77/89 (86.5)	163/214 (76.2)	284/477 (59.5)	462/923 (50.1)	535/1313 (40.7)	540/1647 (32.8)	518/1992 (26.0)
Noninvasive ventilation^d^	6104/6675 (91.4)	2/2 (100)	18/18 (100)	89/89 (100)	214/214 (100)	473/477 (99.2)	910/923 (98.6)	1273/1313 (97.0)	1493/1647 (90.7)	1632/1992 (81.9)
Duration of first course of invasive ventilation, median (IQR), d^e^	3 (2-6)	10 (7-13)	12 (6-18)	5 (2-10)	5 (2-10)	4 (2-8)	4 (2-6)	3 (2-5)	3 (2-5)	3 (2-5)
Any breast milk feeding^f^	6300/8897 (70.8)	4/11 (36.4)	36/57 (63.2)	133/176 (75.6)	255/379 (67.3)	562/748 (75.1)	955/1301 (73.4)	1231/1694 (72.7)	1441/2086 (69.1)	1683/2445 (68.8)
NICU stay, median (IQR), d^g^	46 (35-60)	121 (107-135)	112 (104-125)	94 (81-116)	82 (72-93)	69 (60-82)	60 (50-70)	49 (42-59)	41 (33-51)	34 (27-43)

Abbreviations: CPAP, continuous positive airway pressure; IQR, interquartile range; NICU, neonatal intensive care unit.

^a^ All respiratory treatments were calculated among infants who were admitted to participating NICUs within 7 days after birth, received complete care, and survived to discharge.

^b^ Intravenous dexamethasone.

^c^ Included high-frequency or conventional ventilations via endotracheal tubes.

^d^ Included noninvasive high-frequency ventilation, noninvasive positive pressure ventilation, nasal continuous positive airway pressure, and high-flow oxygen.

^e^ Calculated among infants who received invasive ventilation during NICU hospitalization.

^f^ Calculated among infants for whom enteral feeding was initiated.

^g^ Calculated among infants who received complete care and survived to discharge.

Only 12.1% (1080 of 8923) of infants received continuous positive airway pressure in delivery rooms, with similar rates across different gestational ages, but 26.7% (2378 of 8923) of the infants were intubated in the delivery room (Table 2). Delayed cord clamping was performed in 22.8% (1446 of 6354) of the infants.

Respiratory treatments were evaluated for 6675 infants who were admitted to participating NICUs within 7 days after birth, received complete care, and survived to discharge (Table 2). Overall, 52.7% (3518 of 6675) of infants received surfactant, with the incidence decreasing with increasing GA: 89.9% (98 of 109) at 25 weeks’ GA or less, 75.7% (523 of 691) at 26 to 27 weeks’ GA, 63.1% (1412 of 2236) at 28 to 29 weeks’ GA, and 40.8% (1485 of 3639) at 30 to 31 weeks’ GA. A total of 9.5% (636 of 6675) of infants were prescribed postnatal corticosteroids: 53.2% (58 of 109) at 25 weeks’ GA or less, 25.5% (176 of 691) at 26 to 27 weeks’ GA, 11.9% (267 of 2236) at 28 to 29 weeks’ GA, and 3.7% (136 of 3639) at 30 to 31 weeks’ GA. Overall, 38.8% (2591 of 6675) of infants received invasive ventilation: 64.7% (447 of 691) at 26 to 27 weeks’ GA, 44.6% (997 of 2236) at 28 to 29 weeks’ GA, and 29.1% (1058 of 3638) at 30 to 31 weeks’ GA. Noninvasive respiratory support was provided to 91.4% (6104 of 6675) infants. Among infants with enteral feeds initiated during hospitalization, 70.8% (6300 of 8897) of infants received breast milk.

### Morbidities

A total of 14.5% (1381) VPIs were DAMA and 85.5% (8171) of the infants received complete care in NICUs. Among infants with complete care, oxygen or respiratory support was needed for 29.2% (2379 of 8148) of VPIs at 36 weeks’ corrected GA or at discharge. The incidences of BPD were 74.2% (173 of 233) at 25 weeks’ GA or less, 51.9% (513 of 988) at 26 to 27 weeks’ GA, 33.4% (918 of 2747) at 28 to 29 weeks’ GA, and 19.3% (805 of 4180) at 30 to 31 weeks’ GA. Overall, 10.4% (745 of 7189) of the infants were diagnosed with IVH (grade ≥3) or cystic PVL (Table 3). The incidence of severe brain injury decreased with increasing GA: 26.8% (48 of 179) at 25 weeks’ GA or less, 16.9% (145 of 858) at 26 to 27 weeks’ GA, 11.0% (273 of 2471) at 28 to 29 weeks’ GA, and 7.6% (279 of 3681) at 30 to 31 weeks’ GA. Necrotizing enterocolitis was diagnosed in 4.9% (403 of 8171) of the infants and culture-proven sepsis in 9.4% (764 of 8171). Severe ROP was present in 4.3% (296 of 6851) of the infants.

**Table 3.** Morbidities of Very Preterm Infants (<32 Weeks' Gestation) Admitted to 57 Chinese Neonatal Intensive Care Units and Receiving Complete Care

Variable	Infants, No./total No. (%)									
	Total	Gestational age, wk								
		≤23	24	25	26	27	28	29	30	31
No.	8171	23	52	161	325	663	1185	1570	1917	2275
IVH grade ≥3 or cystic PVL^a^	745/7189 (10.4)	4/11 (36.4)	9/38 (23.7)	35/130 (26.9)	53/286 (18.5)	92/572 (16.1)	129/1059 (12.2)	144/1412 (10.2)	152/1688 (9.0)	127/1993 (6.4)
BPD^b^	2379/8148 (29.2)	21/23 (91.3)	42/52 (80.8)	110/158 (69.6)	204/325 (62.8)	309/663 (46.6)	459/1179 (38.9)	459/1568 (29.3)	411/1915 (21.5)	364/2265 (16.1)
NEC stage ≥2	403/8171 (4.9)	0/23 (0.0)	4/52 (7.7)	17/161 (10.6)	19/325 (5.8)	46/663 (6.9)	67/1185 (5.7)	71/1570 (4.5)	95/1917 (5.0)	84/2275 (3.7)
Sepsis	764/8171 (9.4)	4/23 (17.4)	7/52 (13.5)	31/161 (19.3)	46/325 (14.2)	82/663 (12.4)	152/1185 (12.8)	168/1570 (10.7)	136/1917 (7.1)	138/2275 (6.1)
ROP stage ≥3^c^	296/6851 (4.3)	2/4 (50.0)	11/28 (39.3)	31/118 (26.3)	45/282 (16.0)	45/575 (7.8)	61/1044 (5.8)	39/1410 (2.8)	36/1623 (2.2)	26/1767 (1.5)

Abbreviations: BPD, bronchopulmonary dysplasia; IVH, intraventricular hemorrhage; NEC, necrotizing enterocolitis; NICU, neonatal intensive care unit; PVL, periventricular leukomalacia; ROP, retinopathy of prematurity.

^a^ IVH and PVL were evaluated among infants with neuroimaging results.

^b^ BPD was defined as ventilation or oxygen dependency at 36 weeks’ postmenstrual age or at discharge, transfer, or death if before 36 weeks. Respiratory support data on 23 infants were not available.

^c^ ROP was evaluated among infants with eye examinations.

Morbidities among DAMA infants and all infants admitted are reported in eTable 1 and eTable 2 in the Supplement. DAMA infants had higher incidences of IVH grade 3 or greater or cystic PVL, BPD, and NEC compared with infants who received complete care.

### Survival and Survival Without Major Morbidities

Among 8171 infants who received complete care, the survival rate was 95.4% (7792 of 8171) for all VPIs, 65.6% (155 of 236) at 25 weeks’ GA or less, 89.0% (880 of 988) at 26 to 27 weeks’ GA, 94.9% (2635 of 2755) at 28 to 29 weeks’ GA, and 98.3% (4122 of 4192) at 30 to 31 weeks’ GA (Table 4 and Figure). The rate of survival without major morbidity was 57.2% (4677 of 8171) for all VPIs with complete care, 10.5% (25 of 236) at 25 weeks’ GA or less, 26.8% (265 of 988) at 26 to 27 weeks’ GA, 51.1% (1409 of 2755) at 28 to 29 weeks’ GA, and 69.3% (2904 of 4192) at 30 to 31 weeks’ GA.

**Table 4.** Survival to Discharge for Very Preterm Infants (<32 Weeks' Gestation) Admitted to 57 Chinese Neonatal Intensive Care Units and Receiving Complete Care

Variable	Total	Gestational age, No. (%), wk								
		≤23	24	25	26	27	28	29	30	31
No.	8171	23	52	161	325	663	1185	1570	1917	2275
Survival	7792 (95.4)	5 (21.7)	29 (55.8)	121 (75.2)	285 (87.7)	595 (89.7)	1113 (93.9)	1522 (96.9)	1883 (98.2)	2239 (98.4)
Survival without major morbidity	4677 (57.2)	0 (0.0)	4 (7.7)	21 (13.0)	82 (25.2)	257 (38.8)	533 (45.0)	876 (55.8)	1250 (65.2)	1654 (72.7)

**Figure.**
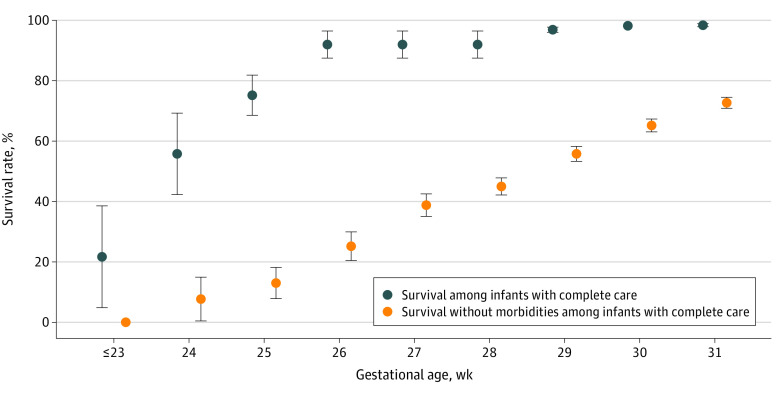
Survival and Survival Without Major Morbidity of Very Preterm Infants (<32 Weeks' Gestation) Admitted to 57 Chinese Neonatal Intensive Care Units and Receiving Complete Care Whiskers indicate 95% CIs.

Among all VPIs admitted, including DAMA infants, 87.6% (8370 of 9552) survived (eTable 3 in the Supplement). A total of 58.1% (803 of 1381) of DAMA infants were expected to die after discharge, accounting for 67.9% (803 of 1182) of all deaths. The survival rate was 67.7% (1115 of 1646) for infants at less than 28 weeks’ GA (15.6% [5 of 32] at ≤23 weeks’ GA, 48.6% [156 of 321] at 24 to 25 weeks’ GA, and 73.8% [954 of 1293] at 26 to 27 weeks’ GA), 88.2% (2828 of 3207) at 28 to 29 weeks’ GA, and 94.2% (4427 of 4699) at 30 to 31 weeks’ GA. Survival without major morbidity was 51.8% (4947 of 9552) for all VPIs, and the rates increased with increasing gestational age: 7.4% (26 of 353) at 25 weeks’ GA or less, 27.2% (352 of 1293) at 26 to 27 weeks’ GA, 46.6% (1494 of 3207) at 28 to 29 weeks’ GA, and 65.4% (3075 of 4699) at 30 to 31 weeks’ GA.

## Discussion

To our knowledge, this study of VPIs admitted to 57 tertiary NICUs throughout China is the first national-level comprehensive assessment of care practices, morbidities, and mortality of VPIs in Chinese NICUs and serves to fill a gap in our knowledge of the current status of neonatal intensive care in China. The study identifies opportunities for improving outcomes of VPIs in China and may enable us to prioritize and target specific areas of care practices for change.

The survival rate of VPIs in our study was 87.6% among all infants admitted to NICUs and 95.4% among infants who received complete care. Our results suggest substantial improvement in survival rates compared with previous reports from China.^16,17,18,19^ Wu et al^16^ reported that the survival rate among 2051 infants with less than 28 weeks’ GA and admitted to NICUs from Guangdong province improved from 36.2% in 2008 to 59.3% in 2017, compared with 67.7% in our study in 2019. Our results also compare favorably with those of Jiang et al,^17^ who reported survival rates among 25 Chinese NICUs for infants with complete care in 2015-2016 of 47% vs 65.6% in our study for infants at 25 weeks’ GA or less, 79% vs 89.0% for infants 26 to 27 weeks’ GA, and 96% vs 97.3% for infants at 28 to 31 weeks’ GA. However, despite the improvement, our outcomes continue to lag behind those of high-income countries. For instance, our current survival rates are comparable only with those of the Eunice Kennedy Shriver National Institute of Child Health and Human Development Neonatal Research Network (NICHD) in 2003-2007.^18^ Compared with the Canadian Neonatal Network in 2018, our survival rates for all NICU admissions were approximately 40% lower for infants at 24 weeks’ GA, 25% lower at 25 to 26 weeks’ GA, 15% lower at 27 to 28 weeks’ GA, and 5% lower at 29 to 31 weeks’ GA.^19^ The difference indicates substantial room for improvement.

One unique problem in China is that a large proportion of infants did not receive complete care and were DAMA. In our study, only 85.5% of VPIs received complete care, which is an improvement over previous reports of less than 80% of VPIs during the past decade.^17,20^ Nevertheless, DAMA remains a factor associated with mortality in VPIs, and we estimated that it accounted for 67.9% (803 of 1182) of deaths in our study. However, DAMA infants also presented higher incidences of morbidities than infants with complete care. Therefore, DAMA infants should be taken into consideration when looking into overall outcomes of preterm infants in China to avoid underestimation. In addition, the high proportion of DAMA in China may be partially due to societal attitudes and lack of social supports for handicapped individuals or to lack of comprehensive health insurance coverage and a high burden of cost to families. Given the high proportion of DAMA and its association with overall outcomes, reduction of DAMA and ensuring that all VPIs receive appropriate care should be one of the priorities for China.

Survival without major morbidity is even more important than survival because it has consequences for long-term developmental outcomes.^21,22,23^ Although overall, 87.6% of infants survived, only 51.8% of infants survived without major morbidity. Among infants with complete care, the discrepancy between survival and survival without major morbidity remained substantial. Although the discrepancy between survival and survival without major morbidity was largest among infants with the lowest gestational age, it remained large among infants with greater gestational age. As survival improves, reducing morbidities and improving quality of life will become increasingly important, and neonatal follow-up, early intervention, and developmental care should be appropriately developed in China.

Among the major morbidities, BPD was the most common in our cohort, occurring in more than one-third of VPIs, and approximately 20% higher than the rates reported by NICHD NICUs in 2008-2012 for each gestational age group.^18^ Addressing BPD through quality improvement measures should be a priority for Chinese NICUs. Our data provide some insights into potential reasons and remedies. For example, only 12.1% of VPIs received continuous positive airway pressure and 26.7% were intubated in the delivery room, suggesting that there may be opportunities for more use of less-invasive strategies in delivery rooms. The second most common morbidity was severe brain injury, which is associated with long-term neurodevelopmental delay.^21,22,23,24^ Interventions such as use of antenatal corticosteroids, maternal rather than infant transport, and elective cesarean delivery for VPIs have been associated with reduced risk of brain injury, and implementation of appropriate practice guidelines could be approaches to reducing severe brain injury.^25^

Another important finding is the high incidence of perinatal risk factors and suboptimal adherence to prenatal care practices that may improve neonatal outcomes. The outborn rate in our study was 36.5%, which is much higher than the 5% to 20% rate reported by most high-income countries,^26^ indicating the need for development of an appropriate perinatal regionalization system, which is lacking in China. Our finding of 75.6% antenatal corticosteroid use is an improvement over previous reports from China (39% in 2008-2012,^27^ 56% in 2013-2014,^28^ and 64% in 2015-2016^17^) but remains much lower than the 80% to 90% rate in high-income countries and indicates a need for better implementation of optimal care guidelines.^18,29^ We also found that the cesarean delivery rates were lower for VPIs in China than in high-income countries, especially among the infants with the lowest GA.^18^ Only 17.1% of infants at 25 weeks’ GA or less and 31.0% of those at 26 to 27 weeks’ GA were born by cesarean delivery in our study. This low rate may reflect parental decision-making, but it may also indicate a need for better communication and discussion between obstetricians, neonatologists, and parents when making active decisions in complex perinatal situations.

### Limitations

Our study had several limitations. The data are from a select group of large tertiary NICUs with the highest level of neonatal care in China and may not be representative of the general population. Our cohort was hospital based rather than population based; in addition, delivery room deaths and infants who were not admitted to NICUs were not included in our study. There was a large predominance of male infants in groups with the lowest gestational ages; one possible cause might be selection bias from our hospital-based design. We made assumptions about mortality of DAMA infants based on predetermined criteria but we did not validate these assumptions by following up the infants after discharge.

## Conclusions

The findings from this study provide national data about outcomes and clinical practices in NICUs and potential strategies for improving care and counseling families in China. Although substantially improved, outcomes of VPIs in China continue to lag behind those of high-income countries. In the future, it may be useful to coordinate efforts in multiple domains, including clinical quality improvement, systems and health services reorganization, strengthening financial and social supports for families, and addressing social determinants of maternal and infant health.

## Notes

Supplement 1. **eFigure.** Map of 58 Participating Hospitals in Chinese Neonatal Network **eTable 1.** Morbidities of Very Preterm Infants (<32 Weeks' Gestation) Admitted to 57 Chinese NICUs and Discharged Against Medical Advice **eTable 2.** Morbidities of All Very Preterm Infants (<32 Weeks' Gestation) Admitted to 57 Chinese NICUs **eTable 3.** Survival to Discharge for All Very Preterm Infants (<32 Weeks' Gestation) Admitted to 57 Chinese NICUs

Supplement 2. **Nonauthor Collaborators.** The Chinese Neonatal Network
