# Supplementary material for: Assessment of Neonatal Intensive Care Unit Practices, Morbidity, and Mortality Among Very Preterm Infants in China
Source: JAMA Netw Open. 2021 Aug 2;4(8):e2118904. doi: 10.1001/jamanetworkopen.2021.18904 (PMC8329742; doi:10.1001/jamanetworkopen.2021.18904)
Supplement: Supplement 1. — eFigure. Map of 58 Participating Hospitals in Chinese Neonatal Network eTable 1. Morbidities of Very Preterm Infants (<32 Weeks' Gestation) Admitted to 57 Chinese NICUs and Discharged Against Medical Advice eTable 2. Morbidities of All Very Preterm Infants (<32 Weeks' Gestation) Admitted to 57 Chinese NICUs eTable 3. Survival to Discharge for All Very Preterm Infants (<32 Weeks' Gestation) Admitted to 57 Chinese NICUs [file jamanetwopen-e2118904-s001.pdf]

Supplementary Online Content

Cao Y, Jiang S, Sun J, et al. Assessment of neonatal intensive care unit practices, morbidity, and mortality among very preterm infants in China. *JAMA Netw Open*. 2021;4(8):e2118904. doi:10.1001/jamanetworkopen.2021.18904

eFigure. Map of 58 Participating Hospitals in Chinese Neonatal Network

eTable 1. Morbidities of Very Preterm Infants (<32 Weeks' Gestation) Admitted to 57 Chinese NICUs and Discharged Against Medical Advice

eTable 2. Morbidities of All Very Preterm Infants (<32 Weeks' Gestation) Admitted to 57 Chinese NICUs

eTable 3. Survival to Discharge for All Very Preterm Infants (<32 Weeks' Gestation) Admitted to 57 Chinese NICUs

This supplementary material has been provided by the authors to give readers additional information about their work.

© 2021 Cao Y et al. *JAMA Network Open.*

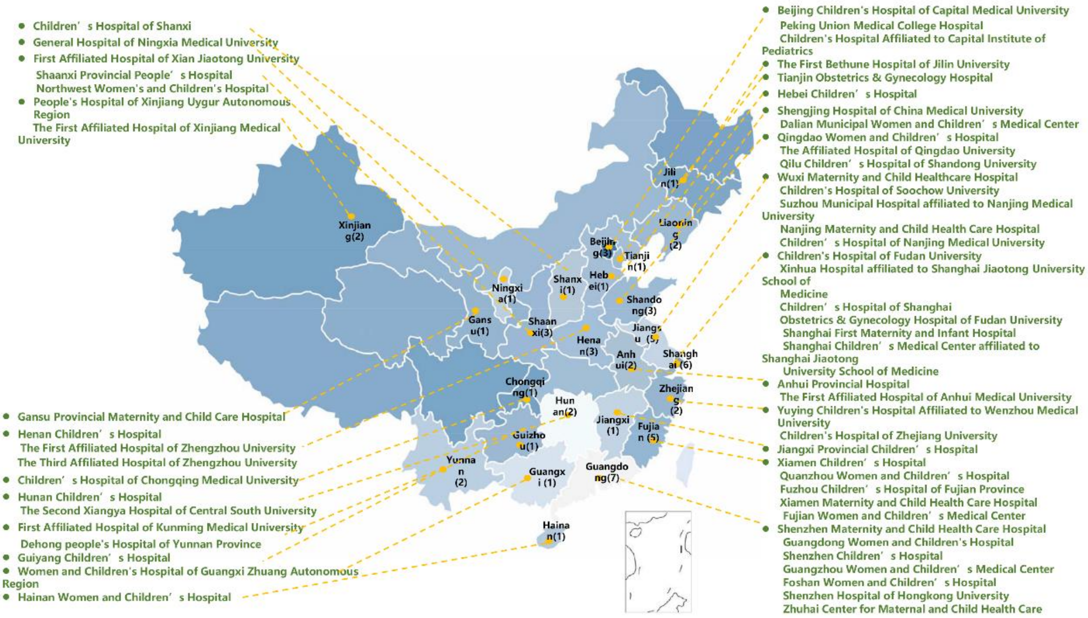

eFigure. Map of 58 participating hospitals in Chinese Neonatal Network

© 2021 Cao Y et al. *JAMA Network Open.*

eTable 1. Morbidities of very preterm infants (<32^{+0} weeks' gestation) admitted to 57 Chinese NICUs and discharged against medical advice

	Total	≤23 weeks	24 weeks	25 weeks	26 weeks	27 weeks	28 weeks	29 weeks	30 weeks	31 weeks
	N=1381	N=9	N=40	N=68	N=126	N=179	N=239	N=213	N=252	N=255
IVH≥grade 3 or cystic PVL[a]	193/840 (23.0%)	2/4 (50.0%)	10/24 (41.7%)	9/33 (27.3%)	32/72 (44.4%)	30/108 (27.8%)	32/138 (23.2%)	27/136 (19.9%)	28/160 (17.5%)	23/165 (13.9%)
BPD[b]	1026/1375 (74.6%)	9/9 (100.0%)	40/40 (100.0%)	65/68 (95.6%)	119/125 (95.2%)	160/179 (89.4%)	192/237 (81.0%)	147/212 (69.3%)	163/251 (64.9%)	131/254 (51.6%)
NEC≥stage 2	93/1381 (6.7%)	0/9 (0.0%)	1/40 (2.5%)	6/68 (8.8%)	2/126 (1.6%)	14/179 (7.8%)	15/239 (6.3%)	17/213 (8.0%)	26/252 (10.3%)	12/255 (4.7%)
Sepsis	107/1381 (7.7%)	0/9 (0.0%)	4/40 (10.0%)	7/68 (10.3%)	12/126 (9.5%)	22/179 (12.3%)	17/239 (7.1%)	20/213 (9.4%)	16/252 (6.3%)	9/255 (3.5%)
ROP≥stage 3[c]	22/500 (4.4%)	N/A	0/5 (0.0%)	0/6 (0.0%)	5/29 (17.2%)	4/59 (6.8%)	5/84 (6.0%)	2/85 (2.4%)	3/117 (2.6%)	3/115 (2.6%)

[a] IVH and PVL was evaluated among infants with neuroimaging results.

[b] BPD was defined as ventilation or oxygen dependency at 36 weeks' postmenstrual age or at discharge/transfer/death if before 36 weeks. Respiratory support on 6 infants were not available.

[c] ROP was evaluated among infants with eye examinations.

Abbreviations: NICU, neonatal intensive care unit; IVH, intraventricular hemorrhage; PVL, periventricular leukomalacia; BPD, bronchopulmonary dysplasia; NEC, necrotizing enterocolitis; ROP, retinopathy of prematurity

© 2021 Cao Y et al. *JAMA Network Open.*

eTable 2. Morbidities of all very preterm infants (<32 weeks' gestation) admitted to 57 Chinese NICUs

	Total	≤23 weeks	24 weeks	25 weeks	26 weeks	27 weeks	28 weeks	29 weeks	30 weeks	31 weeks
	N=9552	N=32	N=92	N=229	N=451	N=842	N=1424	N=1783	N=2169	N=2530
IVH≥grade 3 or cystic PVL[a]	938/8029 (11.7%)	6/15 (40.0%)	19/62 (30.6%)	44/163 (27.0%)	85/358 (23.7%)	122/680 (17.9%)	161/1197 (13.5%)	171/1548 (11.0%)	180/1848 (9.7%)	150/2158 (7.0%)
BPD[b]	3405/9523 (35.8%)	30/32 (93.8%)	82/92 (89.1%)	175/226 (77.4%)	323/450 (71.8%)	469/842 (55.7%)	651/1416 (46.0%)	606/1780 (34.0%)	574/2166 (26.5%)	495/2519 (19.7%)
NEC≥stage 2	496/9552 (5.2%)	0/32 (0%)	5/92 (5.4%)	23/229 (10.0%)	21/451 (4.7%)	60/842 (7.1%)	82/1424 (5.8%)	88/1783 (4.9%)	121/2169 (5.6%)	96/2530 (3.8%)
Sepsis	871/9552 (9.1%)	4/32 (12.5%)	11/92 (12.0%)	38/229 (16.6%)	58/451 (12.9%)	104/842 (12.4%)	169/1424 (11.9%)	188/1783 (10.5%)	152/2169 (7.0%)	147/2530 (5.8%)
ROP≥stage 3[c]	318/7351 (4.3%)	2/4 (50.0%)	11/33 (33.3%)	31/124 (25.0%)	50/311 (16.1%)	49/634 (7.7%)	66/1128 (5.9%)	41/1495 (2.7%)	39/1740 (2.2%)	29/1882 (1.5%)

[a] IVH and PVL was evaluated among infants with neuroimaging results.

[b] BPD was defined as ventilation or oxygen dependency at 36 weeks' postmenstrual age or at discharge/transfer/death if before 36 weeks. Respiratory support on 29 infants were not available.

[c] ROP was evaluated among infants with eye examinations.

Abbreviations: NICU, neonatal intensive care unit; IVH, intraventricular hemorrhage; PVL, periventricular leukomalacia; BPD, bronchopulmonary dysplasia; NEC, necrotizing enterocolitis; ROP, retinopathy of prematurity

© 2021 Cao Y et al. *JAMA Network Open.*

eTable 3. Survival to discharge for all very preterm infants (<32 weeks' gestation) admitted to 57 Chinese NICUs

	Total	≤23 weeks	24 weeks	25 weeks	26 weeks	27 weeks	28 weeks	29 weeks	30 weeks	31 weeks
	N=9552	N=32	N=92	N=229	N=451	N=842	N=1424	N=1783	N=2169	N=2530
Survival	8370 (87.6%)	5 (15.6%)	31 (33.7%)	125 (54.6%)	312 (69.2%)	642 (76.2%)	1206 (84.7%)	1622 (91.0%)	2024 (93.3%)	2403 (95.0%)
Survival without major morbidity	4947 (51.8%)	0 (0.0%)	4 (4.3%)	22 (9.6%)	84 (18.6%)	268 (31.8%)	567 (39.8%)	927 (52.0%)	1316 (60.7%)	1759 (69.5%)

© 2021 Cao Y et al. *JAMA Network Open.*
