# Supplementary material for: Assessment of Neonatal Intensive Care Unit Practices, Morbidity, and Mortality Among Very Preterm Infants in China
Source: JAMA Netw Open. 2021 Aug 2;4(8):e2118904. doi: 10.1001/jamanetworkopen.2021.18904 (PMC8329742; doi:10.1001/jamanetworkopen.2021.18904)
Supplement: Supplement 2. — Nonauthor Collaborators. The Chinese Neonatal Network [file jamanetwopen-e2118904-s002.pdf]

Supplemental Online Content: Nonauthor Collaborators

*Indicates required information. Only first name, last name, and suffix will appear in PubMed.

*Group Name(s): The Chinese Neonatal Network							
***First Name and Middle Initial(s)**	***Last Name**	***Suffix (eg, Jr, III)**	Academic Degrees	Institution	Location (city, state/province, country)	Role or Contribution, eg, chair, principal investigator	Group (if more than 1 Group listed in the byline) and/or Subgroup (eg, Steering Committee)
Falin	Xu		MD	The Third Affiliated Hospital of Zhengzhou University	Zhengzhou, Henan, China	Site investigators	
Xiuying	Tian		MD	Tianjin Obstetrics & Gynecology Hospital	Tianjin, Tianjin, China	Site investigators	
Yong	Ji		MD	Children's Hospital of Shanxi	Taiyuan, Shanxi, China	Site investigators	
Zhankui	Li		MD	Northwest Women's and Children's Hospital	Xi'an, Shaanxi, China	Site investigators	
Jingyun	Shi		MD	Gansu Provincial Maternity and Child Care Hospital	Lanzhou, Gansu, China	Site investigators	
Xindong	Xue		MD	Shengjing Hospital of China Medical University	Shenyang, Liaoning, China	Site investigators	
Chuanzhong	Yang		MD	Shenzhen Maternity and Child Health Care Hospital	Shenzhen, Guangdong, China	Site investigators	
Dongmei	Chen		MD	Quanzhou Women and Children's Hospital	Quanzhou, Fujian, China	Site investigators	
Sannan	Wang		MD	Suzhou Municipal Hospital affiliated to Nanjing Medical University	Suzhou, Jiangsu, China	Site investigators	
Ling	Liu		MD	Guizhou Women and Children's Hospital/Guiyang Children's Hospital	Guiyang, Guizhou, China	Site investigators	
Xirong	Gao		MD	Hunan Children's Hospital	Changsha, Hunan, China	Site investigators	
Changyi	Yang		MD	Fujian Maternity and Child Health Hospital, Affiliated Hospital of Fujian Medical University	Fuzhou, Fujian, China	Site investigators	
Shuping	Han		MD	Nanjing Maternity and Child Health Care Hospital	Nanjing, Jiangsu, China	Site investigators	
Ruobing	Shan		MD	Qingdao Women and Children's Hospital	Qingdao, Shandong, China	Site investigators	
Hong	Jiang		MD	The Affiliated Hospital of Qingdao University	Qingdao, Shandong, China	Site investigators	
Gang	Qiu		MD	Children's Hospital of Shanghai	Shanghai, Shanghai, China	Site investigators	
Qiufen	Wei		MD	Women and Children's Hospital of Guangxi Zhuang Autonomous Region	Xining, Guangxi, China	Site investigators	
Rui	Cheng		MD	Children's Hospital of Nanjing Medical University	Nanjing, Jiangsu, China	Site investigators	
Wenqing	Kang		MD	Henan Children's Hospital	Zhengzhou, Henan, China	Site investigators	
Mingxia	Li		MD	The First Affiliated Hospital of Xinjiang Medical University	Urumqi, Xinjiang, China	Site investigators	
Yiheng	Dai		MD	Foshan Women and Children's Hospital	Foshan, Guangdong, China	Site investigators	
Lili	Wang		MD	The First Affiliated Hospital of Anhui Medical University	Hefei, Anhui, China	Site investigators	
Jiangqin	Liu		MD	Shanghai First Maternity and Infant Hospital	Shanghai, Shanghai, China	Site investigators	
Zhenlang	Lin		MD	Yuying Children's Hospital Affiliated to Wenzhou Medical University	Wenzhou, Zhejiang, China	Site investigators	

Supplemental Online Content: Nonauthor Collaborators

*Indicates required information. Only first name, last name, and suffix will appear in PubMed.

***First Name and Middle Initial(s)**	***Last Name**	***Suffix (eg, Jr, III)**	Academic Degrees	Institution	Location (city, state/province, country)	Role or Contribution, eg, chair, principal investigator	Group (if more than 1 Group listed in the byline) and/or Subgroup (eg, Steering Committee)
Xiuyong	Cheng		MD	The First Affiliated Hospital of Zhengzhou University	Zhengzhou, Henan, China	Site investigators	
Jiahua	Pan		MD	The First Affiliated Hospital of USTC, Division of Life Sciences and Medicine, University of Science and Technology of China	Hefei, Anhui, China	Site investigators	
Qin	Zhang		MD	Shaanxi Provincial People's Hospital	Xi'an, Shaanxi, China	Site investigators	
Xing	Feng		MD	Children's Hospital of Soochow University	Suzhou, Jiangsu, China	Site investigators	
Qin	Zhou		MD	Wuxi Maternity and Child Healthcare Hospital	Wuxi, Jiangsu, China	Site investigators	
Long	Li		MD	People's Hospital of Xinjiang Uygur Autonomous Region	Urumqi, Xinjiang, China	Site investigators	
Pingyang	Chen		MD	The Second Xiangya Hospital of Central South University	Changsha, Hunan, China	Site investigators	
Ling	Yang		MD	Hainan Women and Children's Hospital	Haikou, Hainan, China	Site investigators	
Deyi	Zhuang		MD	Xiamen Children's Hospital	Xiamen, Fujian, China	Site investigators	
Yongjun	Zhang		MD	Xinhua Hospital affiliated to Shanghai Jiao Tong University School of Medicine	Shanghai, Shanghai, China	Site investigators	
Jinxing	Feng		MD	Shenzhen Children's Hospital	Shenzhen, Guangdong, China	Site investigators	
Li	Li		MD	Children's Hospital Affiliated to Capital Institute of Pediatrics	Beijing, Beijing, China	Site investigators	
Xinzhu	Lin		MD	Women and Children's Hospital, School of Medicine, Xiamen university	Xiamen, Fujian, China	Site investigators	
Yinping	Qiu		MD	General Hospital of Ningxia Medical University	Yinchuan, Ningxia, China	Site investigators	
Kun	Liang		MD	First Affiliated Hospital of Kunming Medical University	Kunming, Yunnan, China	Site investigators	
Li	Ma		MD	Hebei Provincial Children's Hospital	Shijiazhuang, Hebei, China	Site investigators	
Liping	Chen		MD	Jiangxi Provincial Children's Hospital	Nanchang, Jiangxi, China	Site investigators	
Liyan	Zhang		MD	Fuzhou Children's Hospital of Fujian Province	Fuzhou, Fujian, China	Site investigators	
Hongxia	Song		MD	First Affiliated Hospital of Xi'an Jiao Tong University	Xi'an, Shaanxi, China	Site investigators	
Zhaoqing	Yin		MD	Dehong people's Hospital of Yunnan Province	Dehong, Yunnan, China	Site investigators	
Huiwen	Huang		MD	Zhuhai Center for Maternal and Child Health Care	Zhuhai, Hainan, China	Site investigators	
Jie	Yang		MD	Guangdong Women and Children's Hospital	Guangzhou, Guangdong, China	Site investigators	
Dong	Li		MD	Dalian Municipal Women and Children's Medical Center	Dalian, Shandong, China	Site investigators	
Guofang	Ding		MD	Peking Union Medical College Hospital	Beijing, Beijing, China	Site investigators	
Jimei	Wang		MD	Obstetrics & Gynecology Hospital of Fudan University	Shanghai, Shanghai, China	Site investigators	
Qianshen	Zhang		MD	Shenzhen Hospital of Hongkong University	Shenzhen, Guangdong, China	Site investigators	
